# Molecular Characterization of *LjABCG1*, an ATP-Binding Cassette Protein in *Lotus japonicus*

**DOI:** 10.1371/journal.pone.0139127

**Published:** 2015-09-29

**Authors:** Akifumi Sugiyama, Shoju Fukuda, Kojiro Takanashi, Miki Yoshioka, Hirofumi Yoshioka, Yoshihiro Narusaka, Mari Narusaka, Mikiko Kojima, Hitoshi Sakakibara, Nobukazu Shitan, Shusei Sato, Satoshi Tabata, Masayoshi Kawaguchi, Kazufumi Yazaki

**Affiliations:** 1 Laboratory of Plant Gene Expression, Research Institute for Sustainable Humanosphere, Kyoto University, Gokasho, Uji, 611–0011, Japan; 2 Laboratory of Defense in Plant-Pathogen Interactions, Graduate School of Bioagricultural Sciences, Nagoya University, Nagoya, 464–8601, Japan; 3 Research Institute for Biological Sciences, Okayama, 7549–1, Yoshikawa, Kaga-gun, Okayama, 716–1241, Japan; 4 RIKEN Center for Sustainable Resource Science, 1-7-22, Suehiro, Tsurumi, Yokohama, 230–0045, Japan; 5 Laboratory of Natural Medicinal Chemistry, Kobe Pharmaceutical University, Kobe, 658–8558, Japan; 6 Graduate School of Life Sciences, Tohoku University, 2-1-1 Katahira, Aoba-ku, Sendai, 980–8577, Japan; 7 Kazusa DNA Research Institute, 2-6-7, Kazusa-Kamatari, Kisarazu, Chiba, 292–0812, Japan; 8 National Institute for Basic Biology, Nishigonaka 38, Myodaiji, Okazaki, 444–8585, Japan; National Taiwan University, TAIWAN

## Abstract

*LjABCG1*, a full-size ABCG subfamily of ATP-binding cassette proteins of a model legume, *Lotus japonicus*, was reported as a gene highly expressed during the early stages of nodulation, but have not been characterized in detail. In this study we showed that the induction of *LjABCG1* expression was remarkable by methyl jasmonate treatment, and reporter gene experiments indicated that *LjABCG1* was strongly expressed in the nodule parenchyma and cell layers adjacent to the root vascular tissue toward the nodule. *LjABCG1* was suggested to be localized at the plasma membrane based on the fractionation of microsomal membranes as well as separation via aqueous two-phase partitioning. The physiological functions of *LjABCG1* in symbiosis and pathogenesis were analyzed in homologous and heterologous systems. *LjABCG1* knock-down *L*. *japonicus* plants did not show clear phenotypic differences in nodule formation, and not in defense against *Pseudomonas syringae*, either. In contrast, when *LjABCG1* was expressed in the Arabidopsis *pdr8-1* mutant, the penetration frequency of *Phytophthora infestans*, a potato late blight pathogen, was significantly reduced in *LjABCG1/pdr8-1* than in *pdr8-1* plants. This finding indicated that *LjABCG1*, at least partially, complemented the phenotype of *pdr8* in Arabidopsis, suggesting the multiple roles of this protein in plant-microbe interactions.

## Introduction

ATP-binding cassette (ABC) proteins, which are ubiquitous in all prokaryotes and eukaryotes, constitute one of the largest families in plants, functioning as transporters, channel regulators, and molecular switches. The full-size ABCG subfamily of ABC proteins is found in plants, fungi, oomycetes, brown algae and slime molds, but not in the genomes of bacteria and animals [1,2] [3]. In addition to being involved in heavy metal tolerance and plant signal molecule transport [4–7], full-size ABCG proteins have been shown to function in response to plant pathogens. NpPDR1 of *Nicotiana plumbaginifolia* was reported responsible for the secretion of an antifungal diterpene compound, sclareol, at the leaf surface [8], and the RNAi-mediated suppression of NpPDR1 rendered transgenic plants susceptible to infection by the gray mold pathogen *Botrytis cinerea* [9]. AtABCG36 (AtPDR8) of *Arabidopsis thaliana* was identified as a gene induced by pathogen attacks and also during the screening of mutants defective in non-host resistance [10,11]. A knock-out mutant of this gene showed a high frequency of penetration by the oomycete pathogen *Phytophthora infestans*, suggesting that AtABCG36 is involved in plant surface resistance to penetration by this pathogen [10].

Various stresses have been reported to induce the expression of genes encoding the full-size ABCG subfamily in plants. For example, NtPDR1 of *N*. *tabacum* was strongly induced by methyl jasmonate (MeJA), yeast extract, and flagellin [12,13]; AtABCG40 (AtPDR12) was induced by salicylic acid, MeJA, ethylene and both compatible and incompatible pathogens [14]; GmPDR12 of soybean (*Glycine max*) was also inducible by salicylic acid and MeJA [15]; and MtABCG10 of *Medicago truncatula* was induced by fungal elicitor and may be involved in defenses against fungal pathogens [16]. These findings suggest that full-size ABCG proteins are employed in defense responses in both monocots and dicots, although the substrates of most of these transporter molecules have not yet been identified.

*Lotus japonicus* is a representative legume plant, which, together with *M*. *truncatula*, is utilized as a model for studies on sophisticated plant responses that result in a symbiotic relationship with rhizobia. Several full-size ABCG proteins of *L*. *japonicus* have been reported induced in response to *rhizobium* infection and to be expressed in nodules [17,18], and tissue-specific microarray analysis showed that these genes were expressed in various parts of nodules [19]. Despite these findings on the expression of full-size ABCG proteins in *L*. *japonicus*, their physiological functions have not yet been characterized. We have therefore investigated *LjABCG1*, a gene highly expressed during the early stages of the nodulation process. The expression of *LjABCG1* in *L*. *japonicus* was characterized in detail, as was phenotypic analysis upon infection by rhizobia and pathogens.

## Materials and Methods

### Plant material and growth conditions

*Lotus japonicus* plants (MG-20) were grown on a mixture of vermiculite and soil in growth chambers with 100 or 120 μmol m^–2^ s^–1^ light in a 17-h-light/7-h-dark cycle at 23°C. For growth under sterile conditions, seeds were surface-sterilized in 0.5% sodium hypochlorite for 20 min, rinsed five times in sterile distilled water and sown on half-strength B5 medium [20] supplemented with 1% sucrose and 0.8% agar. To analyze the regulation of *LjABCG1* by various growth regulators, seeds were sown onto nylon mesh (200 μm pore) over a half-strength B5 medium and grown for 14 days under the same light cycle described above. Roots were subjected to various treatments by gentle transfer of the mesh to new medium containing various chemicals, as described [21]. Treatments were stopped by immediate freezing of seedlings in liquid nitrogen. For inoculation of *M*. *loti*, sterilized seeds were sown on vermiculite supplemented with half-strength NF medium [22]. One-week-old seedlings were inoculated with the symbiotic bacteria, *M*. *loti* strain *Tono*, which had been cultured in YEM medium for two days at 28°C, as described [18]. In order to analyze the induction of *LjABCG1* via shoot-derived signals, MeJA was applied with a cotton ball to the leaves of plants grown in the plant box, as described [14].

### Cloning of *LjABCG1* and vector construction

To isolate *LjABCG1* (chr3.CM0226.110.r2.m) cDNA, the primers 5’-GAGAGTCGACATGGAGGGGGGTGATATATAC-3’ (forward) and 5’-AAGGAAAAAAGCGGCCGCCTAACGTCTTTGGAAGTTGAAG-3’ (reverse), where the underlined non-native sequences represent *Sal* I and *Not* I restriction sites, respectively, were used to PCR amplify cDNA using Phusion High-Fidelity DNA Polymerase (Finnzymes). The PCR product was subcloned into the *Sal* I and *Not* I sites of pENTR1A vector (Invitrogen). This construct served as the entry vector to transfer *LjABCG1* cDNA into the binary destination vector pGWB2 for constitutive expression via the Gateway system (Invitrogen), generating the Pro35S::LjABCG1 construct with a cauliflower mosaic virus 35S promoter.

For RNAi constructs, the primers 5’-GGGGACAAGTTTGTACAAAAAAGCAGGCTGGTGTAAGTGGAGCTGGTAAAA-3’ (forward) and 5’-GGGGACCACTTTGTACAAGAAAGCTGGGTAATACTTGGTTGATGGATGGTG-3’ (reverse), where the underlined positions represent attB1and attB2 sites, respectively, were used in PCR reactions with KOD plus DNA polymerase (TOYOBO). The amplified fragment, which contained the region from nt 2,638 to nt 3,144 of *LjABCG1*, was cloned into pDONR221 (Invitrogen) using BP Clonase (Invitrogen), and subsequently into pGWB80 using LR Clonase.

### Transformation of *L*. *japonicus*

*Agrobacterium tumefaciens*-mediated hypocotyl transformation of *L*. *japonicus* was performed as described [23], with some modification. Briefly, *A*. *tumefaciens* EHA101 was used, and the 6-benzyl aminopurine and α-naphthalene acetic acid concentrations in callus induction and shoot induction media were set at 0.3 μg/ml and 0.1μg/ml, respectively. Hairy root transformation with *Agrobacterium rhizogenes* LBA 1334 was performed as described [24]. This strain was imported on license from Ministry of Agriculture, Forestry and Fisheries of Japan.

### RNA isolation and real time quantitative reverse transcription PCR

Total RNA was isolated with the RNeasy Plant Mini-Kit (Qiagen, Valencia, CA), according to the manufacturer’s instructions. The RNA was reverse transcribed using SuperScript III reverse transcriptase (Invitrogen, CA), followed by incubation with RNase H (Invitrogen, CA). Real-time PCR reactions were performed with the Roter-Gene 3000A (Corbett Research, Australia), using Platinum SYBR Green qPCR SuperMix-UDG (Invitrogen, CA) according to the manufacturers’ instructions. Briefly, each PCR reaction mixture consisted of 10 ng of cDNA template, 5 pmol of each primer, 1 μl of fluorescent probe and 12.5 μl of Platinum Quantitative PCR SuperMix-UDG in a total volume of 25 μl. The amplification protocol consisted of an initial denaturation at 95°C for 10 min, followed by 40 cycles of denaturation at 95°C for 15 sec, annealing at 55°C for 30 sec, and extension at 72°C for 30 sec. The primer sets consisted of 5’GATGGTTGCGTCGCAGTT-3’ (forward) and 5’-CTAACGTCTTTGGAAGTTGAAG-3’ (reverse) for *LjABCG1* and 5’-CAACTGGGACGAYATGGAGA-3’ (forward) and 5’-GAGTCATCTTCTCTCTGTTGGCC-3’ (reverse) for actin.

### Isolation of promoter region and GUS expression analysis

The promoter sequences of *LjABCG1* were isolated by amplification of genomic using PCR primers flanking the regions from nt +3 to –2,003, 5’-AAGCTTGACTTAGAAATGAAGTAGTGGTGGA-3’ (forward) and 5’-TCTAGACATTGTTGCTGCCACCCAGT-3’ (reverse), where the underlined positions represent *Hin*d III and *Xba* I restriction sites, respectively, and KOD-Plus DNA polymerase. The PCR product was inserted upstream of the GUS coding sequence in the pBI101 binary vector to form ProLjABCG1::GUS. Transgenic *Lotus* plants were histochemically stained to detect GUS activity, as described [21,25].

### Inoculation of *P*. *syringae* to *L*. *japonicus*

Inoculation was performed essentially as described in the previous report [26]. *P*. *syringae* pv. *pisi* MAFF730032 was grown at 28°C to the stationary phase in Luria-Bertani medium containing 10 mM MgCl_2_. Bacterial culture was centrifuged and washed with sterilized distilled water. Bacterial cells were then suspended in sterilized water at a concentration of 1 × 10^7^ cells per milliliter, and applied to the roots of 14-day-old plants growing in plant box containing vermiculite supplemented with half-strength NF medium and 1 mM KCl. After 28 days of inoculation, the brownish pigmentation was observed under microscope.

### Transformation of Arabidopsis, infection with *P*. *infestans* and *P*. *syringae*, hormone analysis, and transformation of tobacco BY-2 cells

*Agrobacterium tumefaciens* GV3101 (pMP90) was used for transformation of Arabidopsis via the floral dip method [27], with T_2_ generation plants used for infection assays. *P*. *infestans* race 0 was maintained on tubers of susceptible potatoes and suspensions of Phytophthora zoospores were prepared as described [28]. The zoospore suspension (1×10^5^ ml^–1^) was applied to attached leaves using a lens paper under 95% humidity at 23°C. Trypan blue staining was used to visualize cell death and infection structures in leaves infected with *P*. *infestans*, as described [29]. Stained leaves were observed under a fluorescence microscope (Axio Imager, Carl Zeiss).

Spontaneous rifampicin resistant colonies of *P*. *syringae* pv. maculicola (MAFF302783Rif4) (Psm) were obtained by culturing strain MAFF302783 in King’s B medium containing 100 μg ml-1 of rifampicin [30]. Psm was grown in liquid King’s B medium containing rifampicin (25 μg ml^-1^). Bacteria were harvested by centrifugation, and cell pellets were washed with 10 mM MgSO_4_ and resuspended in 10 mM MgSO_4_ to a concentration of 1×10^8^ cfu ml^-1^ for in planta growth assays. Five-week-old Arabidopsis Col-0 plants, which were susceptible to Psm, and the transformants were used for virulence assays. Plants were inoculated by spraying the leaves with the bacterial suspension and placed in a growth chamber at 100% relative humidity at 22°C (12 h light cycle). Bacterial growth was determined 0 and 3 d after inoculation, and pathogen growth was determined by measuring *rpoD* mRNA by qRT-PCR as described [31].

For plant hormone analysis approximately 100 mg of rosette leaves from both wild-type and transgenic Arabidopsis (5-week-old) were immediately frozen in liquid nitrogen and analyzed as described in the previous report [32]. Sclareol tolerance test was performed using Arabidopsis expressing *LjABCG1* under 35S promoter essentially as described previously [14]. Transformation and maintenance of tobacco BY-2 cells were performed essentially as described in [33].

### Antiserum against *LjABCG1* peptide

An oligopeptide of LjABCG1, representing positions 29 to 42 (n-FSRSSRHEEDDEEAC-c) was conjugated to keyhole limpet hemocyanin, and the conjugate was injected into rabbits according to a standard protocol (Hayashi-Kasei, Osaka, Japan). The serum obtained after the fourth boost was used for immunoblot analysis. This oligopeptide sequence is completely conserved in the ABCG homologue of *L*. *corniculatus*, which was used in membrane fractionation experiments.

### Density gradient fractionation and two-phase partitioning of membranes

Sucrose density gradient fractionation was performed as described [34]. All procedures were performed on ice or at 4°C unless otherwise stated. Large amounts of roots for biochemical analyses were obtained using a super root culture of *Lotus corniculatus*, a close relative of *L*. *japonicus* [35]. Roots (ca. 80 g) were homogenized in 160 mL of ice-cold homogenizing buffer (10% [v/v] glycerol, 0.5% [w/v] polyvinylpolypyrrolidone, 5 mM EDTA, 100 mM Tris-HCl (pH 8.0), 150 mM KCl, 3.3 mM dithiothreitol, 1 mM phenylmethylsulfonyl fluoride). The homogenate was strained through Miracloth (Merck, Rahway, NJ) and centrifuged at 8,000g for 10 min. This step was repeated twice. The supernatant obtained was then centrifuged at 100,000g for 40 min. The pellet was homogenized by 30 strokes of a Dounce glass homogenizer in 1 ml of resuspension buffer (10% [v/v] glycerol, 1 mM EDTA, 10 mM Tris-HCl (pH 7.6)). The suspension was layered over a 20%/30%/40%/50% (w/v) discontinuous sucrose gradient that contained, in addition to sucrose, 10 mM Tris-HCl buffer (pH 7.6), 1 mM DTT, 1 mM EDTA, followed by centrifugation at 100,000g for 120 min. Each fraction recovered from interfaces was resuspended in ca. 35 ml of resuspension buffer, and centrifuged at 100,000g for 40 min. Each pellet was homogenized by 30 strokes of a Dounce homogenizer in a small volume of resuspension buffer supplemented with 10 μg/mL leupeptin, 2 μg/mL aprotinin, 2 μg/mL pepstatin, 1 mM dithiothreitol, and 1 mM phenylmethylsulfonyl fluoride, and stored at -80°C until use.

Two-phase partitioning was performed essentially as described [36]. The plasma membrane enriched fraction was obtained by three-step, aqueous two-phase partitioning in Dextran T500-PEG 3350 in the presence of 5 mM potassium phosphate buffer (pH 7.8): 6.2% (w/w) polymers plus 3 mM KCl. The upper and lower phases were diluted at least 5- and 10-fold, respectively, with resuspension buffer, with the membrane fractions collected by centrifugation at 100,000*g* for 60 min. Each pellet was resuspended in the same buffer supplemented with 10 μg/mL leupeptin, 2 μg/mL aprotinin, 2 μg/mL pepstatin, 1 mM dithiothreitol, and 1 mM phenylmethylsulfonyl fluoride, frozen in liquid nitrogen, and kept at -80°C until use.

For western blot analyses, proteins were denatured in denaturation buffer (10 mM Tris-HCl, pH 8.0, 40 mM DTT, 1 mM EDTA, 10% [w/v] sucrose, 10 μg/mL pyronine Y, 2% [w/v] SDS) for 10 min at 50°C, fractionated on 7% SDS-PAGE, and transferred to an Immobilon polyvinylidene difluoride membrane (Millipore). Membranes were treated with Blocking One (Nacalai Tesque), followed by incubation with primary antibodies and subsequently with secondary horseradish peroxidase-conjugated anti-rabbit antibodies using standard procedures. Bands were visualized by chemiluminescence (Perkin-Elmer). Antisera used for immunodetection were directed against LjABCG1, PM H^+^-ATPase, a tonoplast and endomembrane marker H^+^-PPase, and endoplasmic reticulum BiP from Arabidopsis.

## Results

### Isolation of *LjABCG1* cDNA

**Fig 1:**
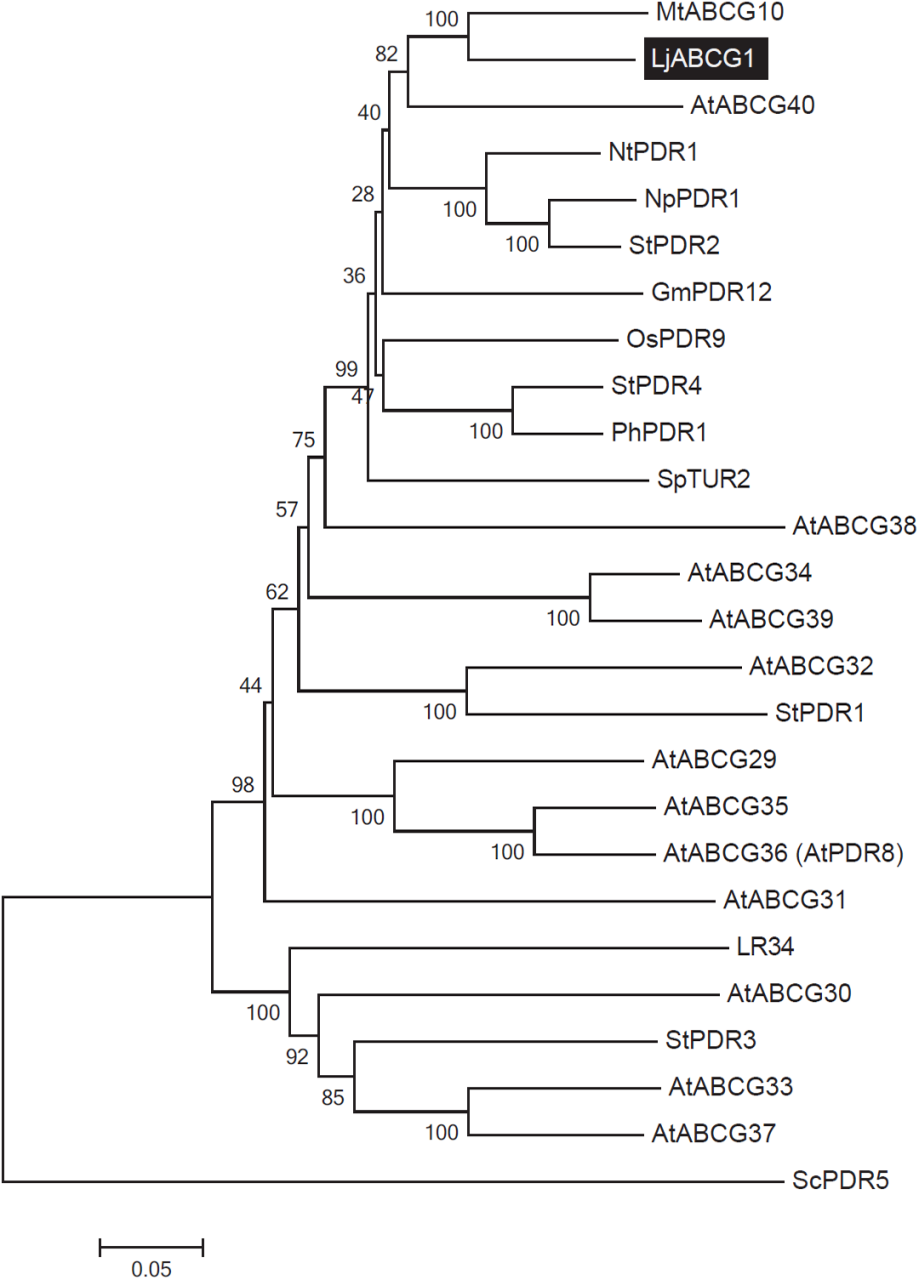
Phylogenetic analysis of plant full-size ABCG transporters. A phylogenetic tree was generated using MEGA 5.0 software [53]. The amino acid sequences of *LjABCG1*, Arabidopsis full-size ABCG transporters and reported full-size ABCG transporters were aligned using the ClustalW program. The Neighbor-joining method was then used to construct a phylogenetic tree with 1,000 bootstrap replications. Boot strap values (max 100) are shown at nodes generating clades. Accession numbers and AGI codes are: ABCG29, At3g16340.1; ABCG30, At4g15230.1; ABCG31, At2g29940.1; ABCG32, At2g26910.1; ABCG33, At2g37280.1; ABCG34, At2g36380.1; ABCG35, At1g15210.1; ABCG36, At1g59870.1; ABCG37, At3g53480.1; ABCG38, At3g30842.1; ABCG39, At1g66950.1; ABCG40, At1g15520.1; GmPDR12, NP_001237697; LR34, ADK62371; MtABCG10, TC184736; OsPDR9, AAQ02685; PhPDR1, AFA43816.1; ScPDR5, CAA99359; StPDR1, AEO22187; StPDR2, AEB65936; StPDR3, AEO22188; StPDR4, AEO22189. At, *Arabidopsis thaliana*; Gm, *Glycine max*; Lj, *Lotus japonicus*; Mt, *Medicago truncatula*; Nt, *Nicotiana tabacum*; Np, *Nicotiana plumbaginifolia*; Os, *Oriza sativa*; Ph, *Petunia hybrida*; Sc, *Saccharomyces cerevisiae*; Sp, *Spirodela polyrhiza*; St, *Solanum tuberosum*.

We previously showed that *LjABCG1* (chr3.CM0226.110.r2.m, formerly called chr3. CM0026.74) belongs to a cluster of full-size ABCG transporter genes, characteristic of the *Lotus* genome, and that its expression was up-regulated in the nodulated roots of mature plants [18]. cDNA array analysis revealed that *LjABCG1* is induced during an early stage of nodulation [17], and tissue-specific expression analysis revealed that *LjABCG1* is expressed mostly in the nodule infection zone and nodule parenchyma [19]. In the *Lotus* genome (http://www.kazusa.or.jp/lotus/), we found that this gene consists of 22 exons and 21 introns (S1 Fig). Its full-length cDNA was isolated by RT-PCR from seedlings and found to encode a 1433-amino acid protein with a predicted molecular weight of 163 kDa (AB935510). LjABCG1 contained two sets of highly conserved Walker A and B boxes, which are involved in ATP and Mg^2+^ binding, respectively, an additional ABC signature between these Walker boxes, and a PDR signature that is conserved among plant full-size ABCG proteins [37]. An amino acid sequence comparison using BLAST revealed the high degree of identify between LjABCG1 and full-size G-type ABC proteins from other plants, including MtABCG10 (79%), NpPDR1 (72%), AtABCG40 (71%), GmPDR12 (68%), SpTUR2 (67%), and AtABCG36 (57%) (Fig 1).

### Expression analysis of *LjABCG1*

**Fig 2:**
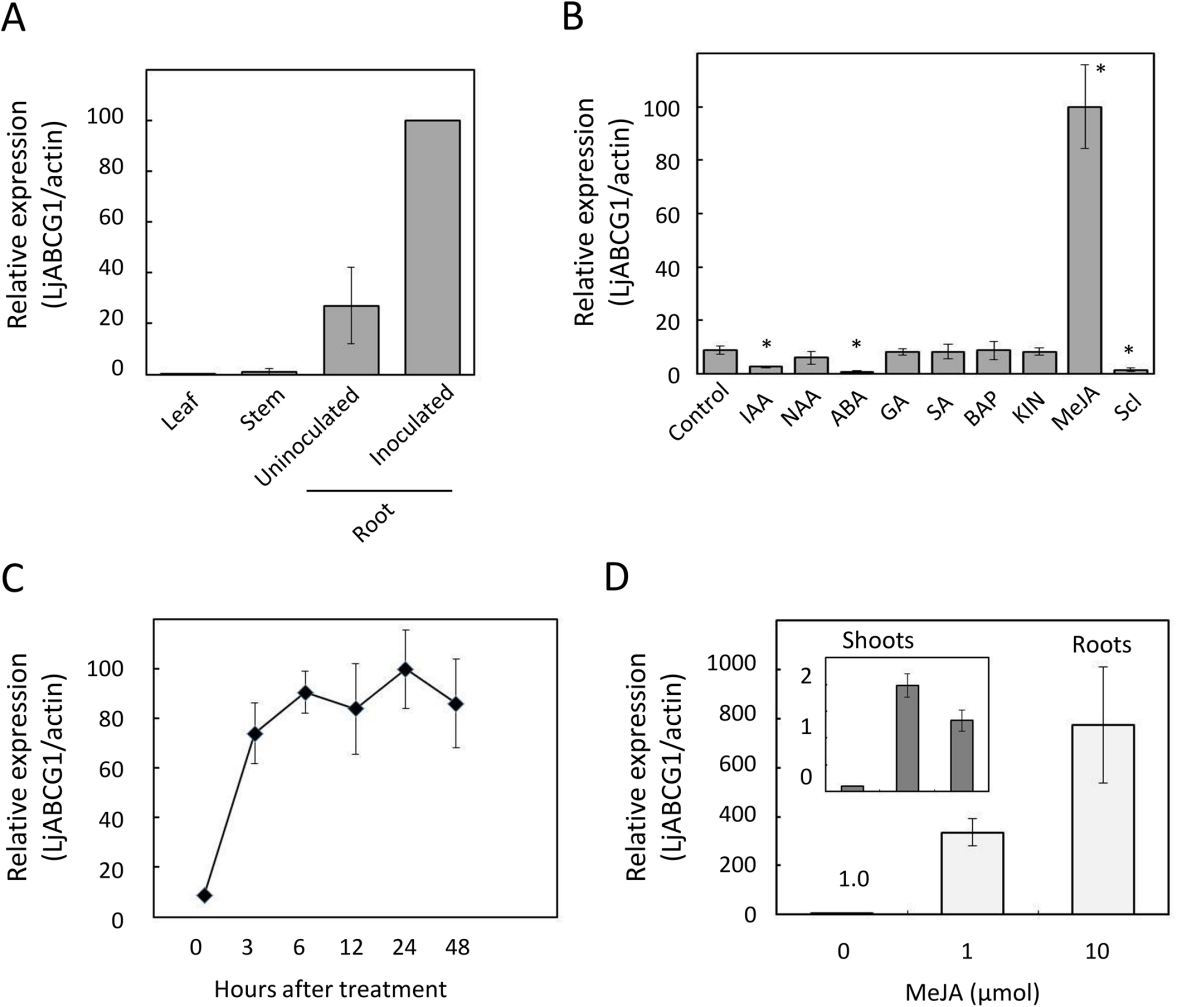
Expression profile of *LjABCG1* in *L*. *japonicus*. (A) Expression of *LjABCG*1 in various tissues. Leaves, stems and inoculated roots were obtained from plants inoculated with *M*. *loti*. (B) Response of *LjABCG1* expression to various treatments. Two-week-old seedlings were treated for 24 h with 10 μM indole-3-acetic acid (IAA), 10 μM 1-naphthaleneacetic acid (NAA), 10 μM abscisic acid (ABA), 10 μM gibberellic acid (GA_3_), 100 μM salicylic acid (SA), 100 μM methyl jasmonate (JA), or 100 μM sclareol (Scl). There chemicals were applied into the medium at the final concentration described above. Significant difference was based on one-way ANOVA with Tukey's HSD test. Asterisks indicate significant differences from control (p<0.05). (C) Time course of expression of *LjABCG1* after treatment with 100 μM MeJA. (D) Response of *LjABCG1* expression to MeJA applied to shoots. A cotton ball containing MeJA was placed onto the wall of an air-tight plant box. After 24 hours, shoots and roots were frozen in liquid nitrogen until RNA preparation. Data represent the means ± S.D. of three replicates.

In *L*. *japonicus*, *LjABCG1* was expressed both in uninfected and infected roots bearing nodules, but its level of expression was very low in aerial parts, including leaves and stems (Fig 2A). The effects of various compounds, including plant hormones, on *LjABCG1* expression were assayed by quantitative real-time PCR in two-week-old seedlings. *LjABCG1* expression was strongly up-regulated by treatment with MeJA, but not by salicylic acid or cytokinins (Fig 2B). The plant hormones indole-3-acetic acid and abscisic acid significantly down-regulated *LjABCG1* expression (Fig 2B). Sclareol, which strongly induced the expression of *NpPDR1*in *Nicotiana plumbaginifolia* and *SpTUR2* in *Spirodela polyrrhiza*, down-regulated the expression of *LjABCG1* in *L*. *japonicus* (Fig 2B).

To further refine the response of *LjABCG1* to MeJA, *LjABCG1* expression in response to MeJA was analyzed over time in two-week-old seedlings. Quantitative real-time PCR showed that *LjABCG1* expression increased about 10-fold within 3 hours, with this high level maintained for up to 48 hours after MeJA treatment (Fig 2C). Because MeJA applied to shoots reportedly inhibits nodulation in *L*. *japonicus* [38] and because JA conjugated with isoleucine was shown to be involved in the regulation of nodulation from shoots [39], the effects of shoot-applied MeJA on the expression of *LjABCG1* in roots was also analyzed. MeJA applied to shoots of *L*. *japonicus* enhanced the expression of *LjABCG1* up to 800-fold in roots and 20-fold in shoots (Fig 2D).

**Fig 3:**
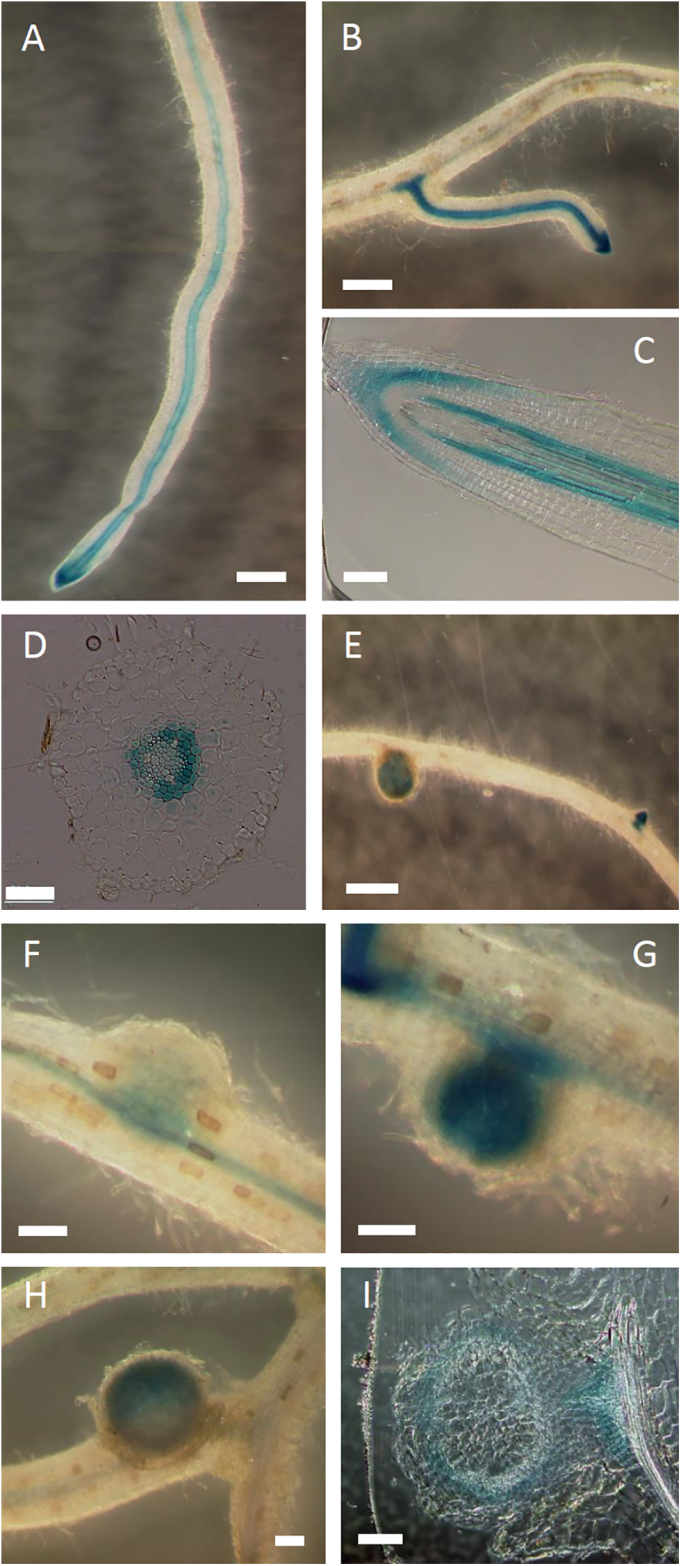
GUS staining of *ProLjABCG1*:*GUS* transformants infected with *M*. *loti*. The transgenic hairy roots were stained with X-Gluc for 24 hours. (A, B) GUS activity in (A) roots and (B) lateral roots. (C) Longitudinal section of a root. (D) Cross-section of a root. (E) Root with a nodule and lateral root primordia. (F-H) Nodules of different stages from F (young) to H (mature). (I) Section of a nodule. Bars = 500 μm (A, B, E), 100 μm (C, F-I), and 50 μm (D).

To analyze the tissue-specific expression pattern of *LjABCG1*, wild-type plants were transformed with Pro_LjABCG1_:GUS, consisting of a 2.0-kb sequence upstream of the LjABCG1 start site fused to a ß-glucuronidase (GUS) reporter gene by the *Agrobacterium rhizogenes*-mediated hairy root transformation method. Hairy roots were histochemically stained with 5-bromo-4-chloro-3-indolyl-ß-glucuronic acid (X-Gluc). GUS expression analysis showed that *LjABCG1* was strongly expressed in steles of lateral roots and young parts of primary roots, especially within a few centimeters of the tips (Fig 3A and 3B), where root cells can be infected by rhizobia [40]. Using cross-sections and longitudinal sections, we observed GUS staining in the root cap (Fig 3C) and in the phloem, pericycle and endodermis (Fig 3D). Strong expression was also observed in the lateral root primordia (Fig 3E). *LjABCG1* is highly expressed in nodules (Fig 3E–3H), and especially strong GUS activity was observed in developing nodules (Fig 3G). Using sections prepared from nodules, we found that *LjABCG1* was not expressed in the infection zone but was expressed in the nodule parenchyma and cell layers adjacent to the root vascular tissue toward the nodule (Fig 3I).

### Subcellular localization of *LjABCG1*

**Fig 4:**
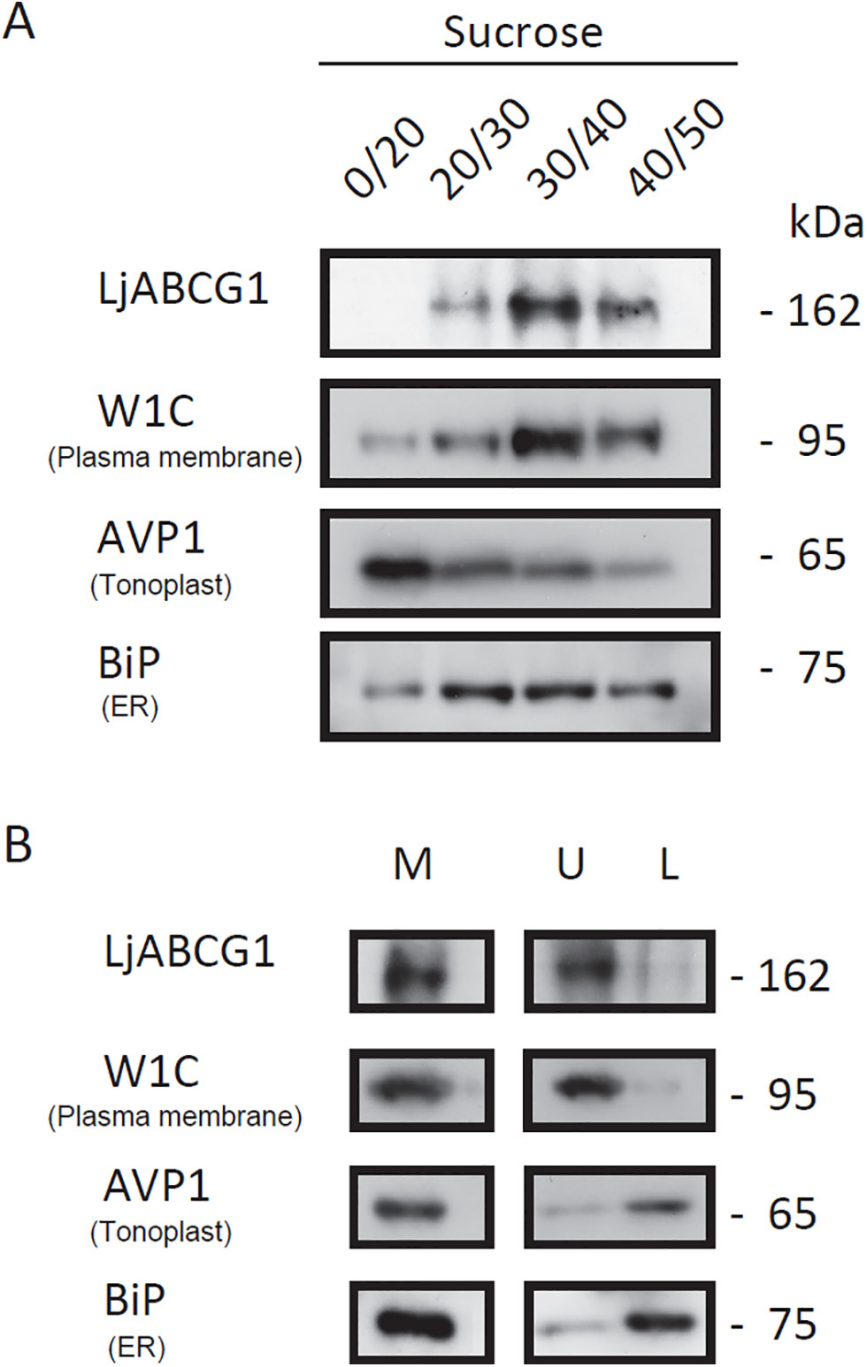
Subcellular localization. (A) Total microsomes from super roots were fractionated on a discontinuous sucrose gradient consisting of 20%, 30%, 40%, and 50% (w/v) sucrose. Membrane fractions were collected from the interfaces between different sucrose concentrations and analyzed via SDS-PAGE and western blotting. Blots were probed with antisera to *LjABCG1*, PM H^+^-ATPase (W1C), tonoplast and endomembrane H^+^-PPase (AVP1), and endoplasmic reticulum BiP (BiP). (B) Two-phase separation of plasma membrane. Microsomal membranes (M) from super roots were fractionated by the aqueous two-phase portioning method, with plasma membranes enriched in the upper phase (U) and other intracellular membranes remaining in the lower phase (L). Proteins from each fraction were blotted and probed with antisera to *LjABCG1*, PM H^+^-ATPase (W1C), tonoplast and endomembrane H^+^-PPase (AVP1), and endoplasmic reticulum BiP (BiP).

We raised peptide antibodies to detect LjABCG1 protein. To confirm the specificity of this antibody, western blot analysis was done using tobacco BY-2 cell expressing LjABCG1, in which a band ca. at 160 kDa of theoretical size was observed in LjABCG1-expressing cells but not in BY-2 cell (S2 Fig). To assess the subcellular localization of LjABCG1, both sucrose density gradient ultrafractionation and aqueous two-phase partitioning system were performed, followed by western blot analysis using antibody against LjABCG1 and root culture of *Lotus corniculatus*, a close relative of *L*. *japonicus* [35]. *L*. *corniculatus* was used for these experiments because we needed an appreciable amount of root mass for microsome fractionation and the root cultures of these plants grow rapidly. The amino acid sequence used to raise the antibody is conserved in LjABCG1 homologue in *L*. *corniculatus*. Sucrose density gradient fractionation of microsomal membranes of *Lotus* roots, followed by western blot analyses with antibody to LjABCG1, showed the presence of this protein in multiple fractions (Fig 4A). The fractionation pattern of LjABCG1 showed a greater correlation with that of the plasma membrane H^+^-ATPase (W1C) than with that of the endoplasmic reticulum marker lumenal binding protein (BiP) or the tonoplast and endomembrane marker H^+^-pyrophosphatase (AVP1) (Fig 4A). Microsomal membranes of *Lotus* root tissues were also separated via aqueous two-phase partitioning with 6.2% (w/w) Dextran T500-PEG 3350. Western gel blot analysis showed that LjABCG1 was found predominantly in the plasma membrane-enriched upper phase, along with the plasma membrane H^+^-ATPase (W1C) (Fig 4B). Taken together, these results suggested that LjABCG1 localizes at the plasma membrane of *L*. *japonicus* root.

### Knock-down of *LjABCG1* in *L*. *japonicus*

To analyze the physiological function of LjABCG1, we prepared RNAi knock-down plants. A construct encoding hairpin-RNA for *LjABCG1* under the control of the CaMV35S promoter was transferred into *L*. *japonicus* MG-20. We obtained more than 20 independent transgenic plants. These transgenic lines were sufficiently fertile and showed no strong phenotype when grown on soil. For detailed analyses, we employed T_2_ progeny that inherited the transgene. Total RNA was isolated from roots and nodules, and *LjABCG1* transcripts were assayed by quantitative real-time RT-PCR. The estimated amounts of *LjABCG1* transcripts were significantly lower in representative transgenic plants than in wild-type plants. There were, however, no clear phenotypic differences in nodule formation and root growth (S3 Fig, Fig 3A–3D). We also performed RNAi-mediated silencing of *LjABCG1* in hairy roots with *Agrobacterium rhizogenes*. In this transformation, we used the pHKN29 vector [24], which contains a marker gene encoding green fluorescent protein (GFP), enabling transformed hairy roots to be distinguished from untransformed roots by fluorescence microscopy. Three weeks after inoculation with *M*. *loti*, GFP-expressing nodules on transgenic hairy roots were counted. The number of nodules did not differ significantly different between control and RNAi roots (data not shown).

We then analyzed the role of LjABCG1 in defense against pathogens. Roots of both wild-type and RNAi plants were infected with *P*. *syringae* pv. *pisi* MAFF730032, which was isolated from *Pisum sativum* stem. *P*. *syringae* pv. *pisi* causes bacterial blight in pea, but did not cause clear disease symptoms in *L*. *japonicus*. Because *bona fide* root pathogen of *L*. *japonicus* has not been isolated [41], the brownish pigmentation in the meristematic region caused by this strain was used as an indicator in pathogenesis [26]. Microscopic observation of roots showed no clear differences between wild-type and RNAi roots in the brownish pigmentation in the meristematic region upon the infection of *P*. *syringae* (S4 Fig).

### Complementation of *abcg36* (*pdr8*) phenotype by over-expressing *LjABCG1* in Arabidopsis

**Fig 5:**
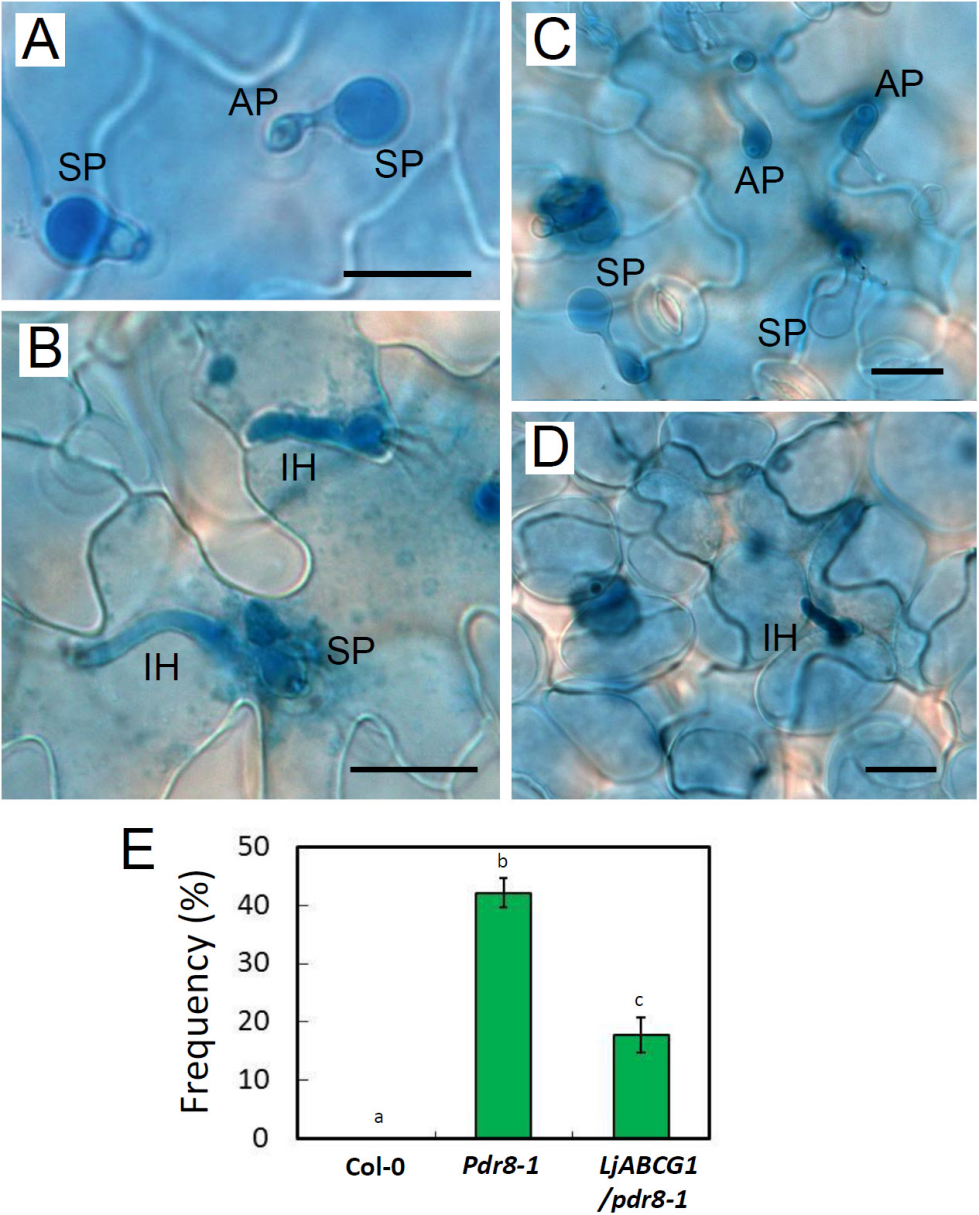
Arabidopsis plants inoculated with *P*. *infestans*. Leaves stained with trypan blue were observed under a microscope 24 h after inoculation with *P*. *infestans*; (A) Col-0, (B) *pdr8-1*, (C) *LjABCG1/pdr8-1*. (D) Beneath image of C. (A-D) SP, spore; AP, appressorium; IH, infection hyphae. Bars = 20 μm. (E) Frequency of penetration of *P*. *infestans* into leaf epidermal cells. Different letters indicate significant differences (p<0.05) in one-way ANOVA with Tukey's HSD test.

We investigated whether LjABCG1 is involved in defense against pathogen using a heterologous system [42]. We employed Arabidopsis *atabcg36* (*pdr8*) mutants, which are defective in resistance to penetration by *P*. *infestans*, with a complementation test performed to determine whether LjABCG1 could complement this defect [10]. *LjABCG1* was expressed on a *pdr8-1* mutant background, and T_2_ transgenic plants expressing *LjABCG1* was used for the infection assays (S5 Fig). Leaves were inoculated with the inappropriate pathogen *P*. *infestans* [28], with cell death and infection structures in the leaves monitored by trypan blue staining. Successful penetration was not observed in wild-type plants (Fig 5A), but infection hyphae were observed in the leaf epidermal cells of *pdr8-1* mutants (Fig 5B). Although infection hyphae of *P*. *infestans* were observed in *LjABCG1/pdr8-1* plants (Fig 5C and 5D), the frequency of penetration was significantly reduced (Fig 5E). Twenty-four h after inoculation, granular cytoplasm was observed in the penetrated epidermal cells, with infection hyphae restricted to these cells (Fig 5C and 5D). These findings suggested that hypersensitive response (HR)-like cell death occurred in response to the pathogen attack, although trypan blue staining of the responding cells was not observed under our experimental conditions. Infection assays were also performed with *P*. *syringae* pv. maculicola, but no difference was observed in *LjABCG1/pdr8-1* plants (S6 Fig). These results suggested that LjABCG1 partially complemented the phenotype of *pdr8* in Arabidopsis.

## Discussion

Transporters play important roles in both symbiotic and pathogenic plant-microbe interactions [43–45]. In particular, full-size ABCG subfamily members have been shown to function in defense against pathogens [8,10,11,13,16]. To date, however, the involvement of this transporter family in symbiotic interactions with rhizobia and in defensive interactions with pathogens has not been compared. To assess the possible involvement of full-size ABCG transporters in nodulation, we focused on LjABCG1, a member of the ABCG subfamily of proteins, strongly expressed in nodules of *L*. *japonicus*. Expression analysis suggested that this protein is involved in pathogenesis rather than in symbiosis. For example, *LjABCG1* was induced during the early stage of nodulation, at the same time that defense-related genes, such as those encoding fungal endoglucanase inhibitor protein and phenylalanine ammonia-lyase, are also induced [17]. We also found that *LjABCG1* was highly induced by MeJA, which is frequently involved in plant defenses and induces the expression of previously characterized ABCG proteins relevant for defenses against pathogens (Fig 2). However, LjABCG1 may not necessarily be involved in a general pathogen-related defense responses, because the root transcriptomic analysis revealed that *LjABCG1* was not induced upon treatment of pathogenic fungal exudates [46]. Shoot-applied MeJA highly induced the expression of *LjABCG1* in roots, suggesting that shoot-derived signals were involved in the induction of *LjABCG1* expression in roots. Jasmonate signaling is correlated with the root exudation in Arabidopsis [47], but our preliminary investigation did not reveal the changes of root exudates upon MeJA application in *L*. *japonicus* (data not shown).

Analysis of subcellular localization of this G-type ABC transporter was performed using root culture of *Lotus corniculatus*, a close relative of *L*. *japonicus* [35]. Sucrose density gradient fractionation of microsomal membranes of *Lotus* roots as well as separation via aqueous two-phase partitioning, followed by western gel blot analysis suggested that LjABCG1 was localized at the plasma membrane (Fig 4). This membrane localization was similar to all other plant full-size ABCG transporters characterized to date, indicating that LjABCG1 mediates the secretion of yet unidentified metabolites to the apoplast. Identifying the transport substrates may help clarify the detailed physiological function of transporters. To date, however, only a few substrates have been identified for these pathogenesis-related ABCG members. Phytoalexin and flavonoids have been suggested as the putative substrates of AtABCG36 and MtABCG10, respectively [10,11,16]; indole-3-butyric acid and abscisic acid were reported as the substrate of AtABCG36 and AtABCG40, respectively [48]. Abscisic acid was suggested to be a negative regulator through regulating the Nod factor signaling and cytokinin induction of nodule development [49], but the transport and perception of abscisic acid in nodule are not yet characterized. Diterpenes such as sclareol was identified as physiological substrates related to defense mechanism for these ABCG transporters in *Nicotiana* spp. [8,13]. In addition, AtABCG40 and SpTUR2, an ABCG member in *Spirodela*, have been suggested to transport sclareol based on the sclareol tolerance test in Arabidopsis [14,50]. Because LjABCG1 share high amino acid identity with these proteins, we also tested the sclareol tolerance using Arabidopsis over-expressing *LjABCG1*. However, no effect on plant tolerance to sclareol was observed (S7 Fig).

To investigate the physiological function of LjABCG1 in *L*. *japonicus*, RNAi-mediated knock-down of *LjABCG1* was performed. Several transgenic *L*. *japonicus* plants with reduced *LjABCG1* expression were obtained, but they did not reveal clear phenotypic differences in root growth and nodule formation (S3 Fig) nor in defense against *P*. *syringae* (S4 Fig). This may be due to the functional redundancy of ABC transporter genes, as suggested for other ABC transporters [51]. Conversely, the remaining 20% of gene expression may be sufficient to support the physiological function of LjABCG1. In the latter case, complete knock-out will likely alter the plant phenotype. Transgenic plants overexpressing LjABCG1 were also generated, but there was no phenotypic difference in nodulation either (S8 Fig).

To test whether LjABCG1 is involved in defense responses against oomycetes pathogens, *LjABCG1* was expressed in the Arabidopsis *abcg36* (*pdr8*) mutant, in which resistance to penetration by the inappropriate pathogen *P*. *infestans* is impaired [10]. LjABCG1 partially complemented the phenotype of the mutants (Fig 5). This result suggests that 1) LjABCG1 transports Arabidopsis endogenous metabolites such as camalexin and glucosinolates or toxic compounds of pathogens in Arabidopsis upon infection or 2) that the overexpression of LjABCG1 disturbed cellular homeostasis in Arabidopsis and the alleviation is an indirect effect. The latter possibility was tested by analyzing plant hormones in *pdr8-1* as well as *LjABCG1/pdr8-1*. There was no significant difference in the analyzed plant hormones among these plants (S1 Table), suggesting that the cellular homeostasis was not strongly disturbed by the ectopic expression of LjABCG1.

Although it is speculative at this point to suggest potential substrates in *L*. *japonicus* based on the results in Arabidopsis, possible target for further investigation would be flavonoid phytoalexin such as medicarpin and vestitol [52], because LjABCG1 is highly expressed in nodule parenchyma, where a set of genes for flavonoid biosynthesis highly expressed [19]. Further studies using pathogens against *L*. *japonicus* is warranted to figure out the physiological function of LjABCG1 in nodule defense; however, no robust pathosystem for *L*. *japonicus* has not been described to date, especially for fungal and oomycetes pathogens, for which phytoalexin plays important roles in defense (Glazebrook et al., 1997; Bednarek et al., 2009); therefore, screening for a pathogen infecting the roots and nodule of this legume plants is prerequisite to investigate the function of LjABCG1 in defense.

## Supporting Information

S1 Fig Exon-intron structure and putative membrane topology of LjABCG1. (TIF)

S2 Fig Expression of LjABCG1 in BY-2 cells. (TIF)

S3 Fig Phenotypes of *L*. *japonicus* transformed with *LjABCG1*-RNAi constructs. Plants were grown on vermiculites supplemented with nitrogen-free medium and inoculated with *M*. *loti*. (A) Transcript levels in T_2_ transgenic plants (#1 to #4) were analyzed by real time PCR, using four-week-old roots inoculated with *M*. *loti*. Data represent the means ± S.D. of triplicate determinations. (B) Number of nodules on wild-type and transgenic plants. Values are the means ± S.D. (n > 20). (C) Root lengths of wild-type and transgenic plants. Values are the means ± S.D. (n > 20) (D) Representative picture of two-week old RNAi plants.
(TIF)

S4 Fig *L*. *japonicus* inoculated with *P*. *syringae* pv. *pisi*. Roots of wild-type (A) and RNAi (B) plants without inoculation, and roots of wild-type (C, E) and RNAi (D, F) plants at 28 days after inoculation by *P*. *syringae* pv. *pisi*. Bars = 100 μm
(TIF)

S5 Fig Gene expression analysis in Arabidopsis. Expression of *LjABCG1*, *pdr8* of Arabidopsis, hygromycin resistance gene, and actin was analyzed by RT-PCR, using cDNA prepared from leaves of wild-type and transgenic plants.
(TIF)

S6 Fig Relative expression of *rpoD* gene in Arabidopsis. Five-week-old *Arabidopsis* plants Col-0 (susceptible to *Psm*) and the transformants were inoculated by spraying the leaves with the bacterial suspension (1×10^8^ cfu ml^-1^). Leaves were harvested at 0 and 3 dpi (black columns), and total RNA was isolated. Pathogen growth was determined by measuring *rpoD* mRNA by qRT-PCR. Bars indicate means ± S.E. Leaves infected with *Psm* developed chlorotic lesions at 3 dpi. No *rpoD* transcripts were detectable at 0 dpi.
(TIF)

S7 Fig Sclareol tolerance in Arabidopsis. Root length of wild-type and transgenic plants expressing LjABCG1. Values are the means ± S.D. (n = 10).
(TIF)

S8 Fig Nodule formation on transgenic *L*. *japonicus* overexpressing LjABCG1. Number of nodules on wild-type and transgenic plants. Values are the means ± S.D. (n > 10).
(TIF)

S1 Table Plant hormone analysis in Arabidopsis leaves. (DOCX)
