# Supplementary figures and images for: Molecular Characterization of LjABCG1, an ATP-Binding Cassette Protein in Lotus japonicus
Source: PLoS One. 2015 Sep 29;10(9):e0139127. doi: 10.1371/journal.pone.0139127 (PMC4587964; doi:10.1371/journal.pone.0139127)

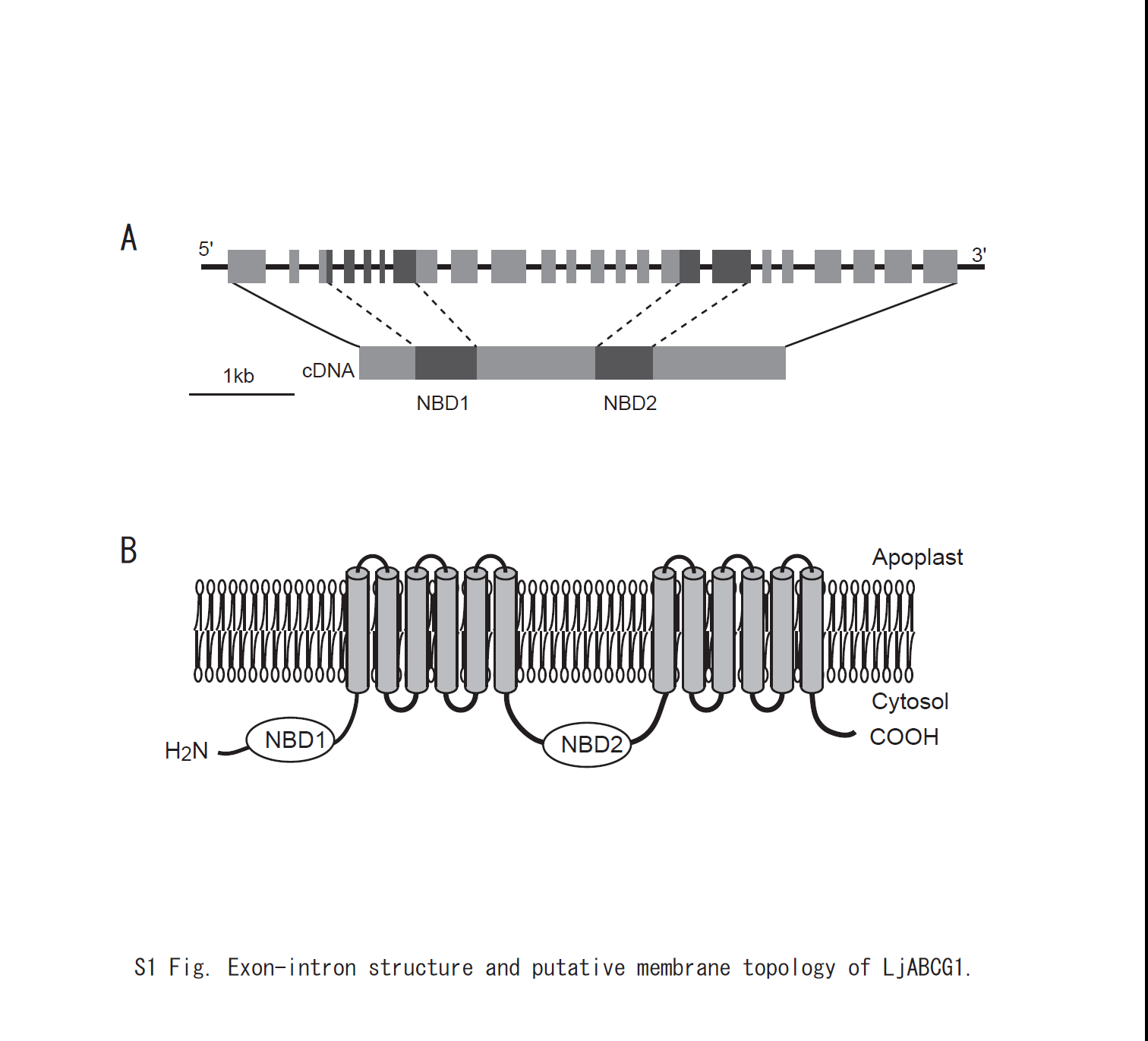

Supplement: S1 Fig — (TIF) [file pone.0139127.s001.tif]

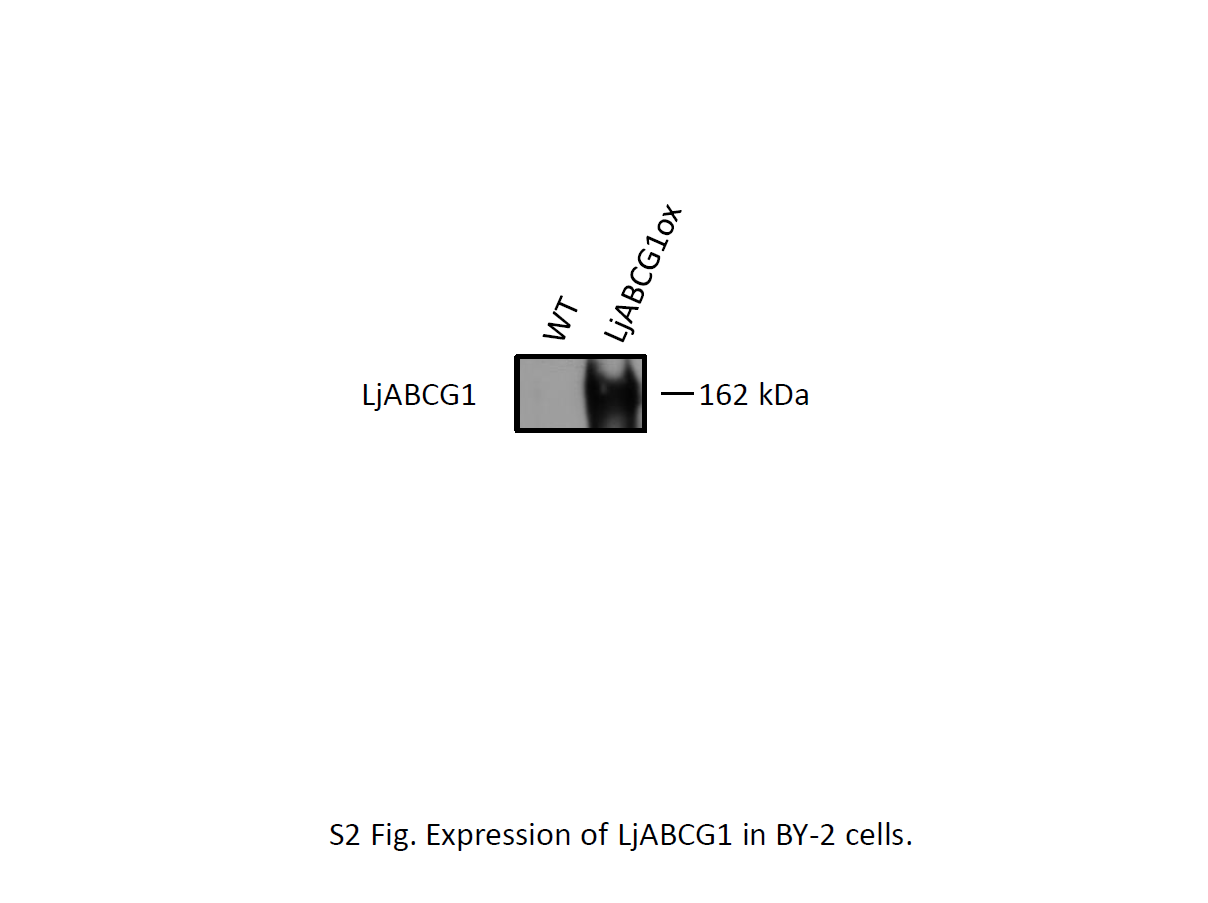

Supplement: S2 Fig — (TIF) [file pone.0139127.s002.tif]

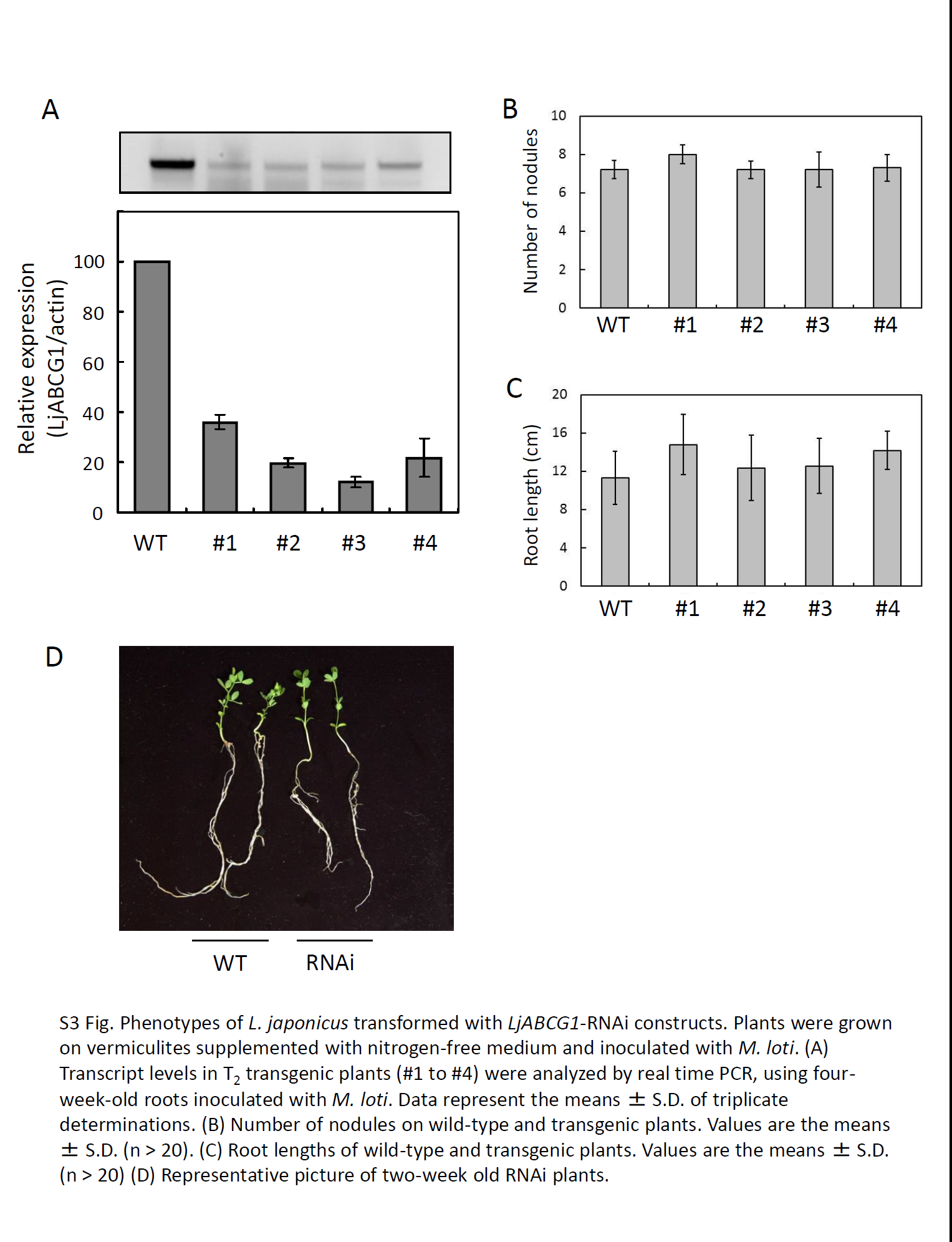

Supplement: S3 Fig — Plants were grown on vermiculites supplemented with nitrogen-free medium and inoculated with M. loti. (A) Transcript levels in T2 transgenic plants (#1 to #4) were analyzed by real time PCR, using four-week-old roots inoculated with M. loti. Data represent the means ± S.D. of triplicate determinations. (B) Number of nodules on wild-type and transgenic plants. Values are the means ± S.D. (n > 20). (C) Root lengths of wild-type and transgenic plants. Values are the means ± S.D. (n > 20) (D) Representative picture of two-week old RNAi plants. (TIF) [file pone.0139127.s003.tif]

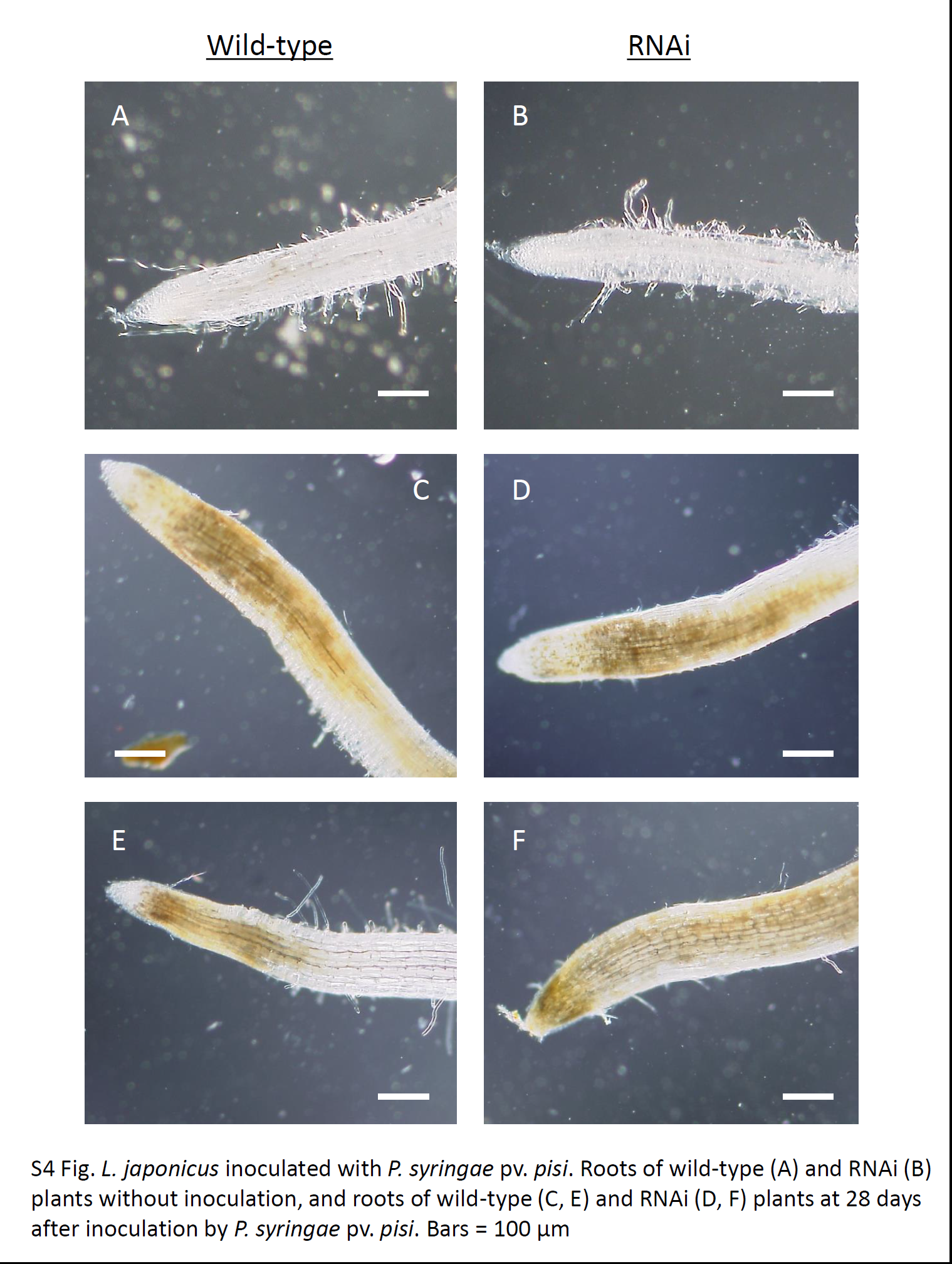

Supplement: S4 Fig — Roots of wild-type (A) and RNAi (B) plants without inoculation, and roots of wild-type (C, E) and RNAi (D, F) plants at 28 days after inoculation by P. syringae pv. pisi. Bars = 100 μm (TIF) [file pone.0139127.s004.tif]

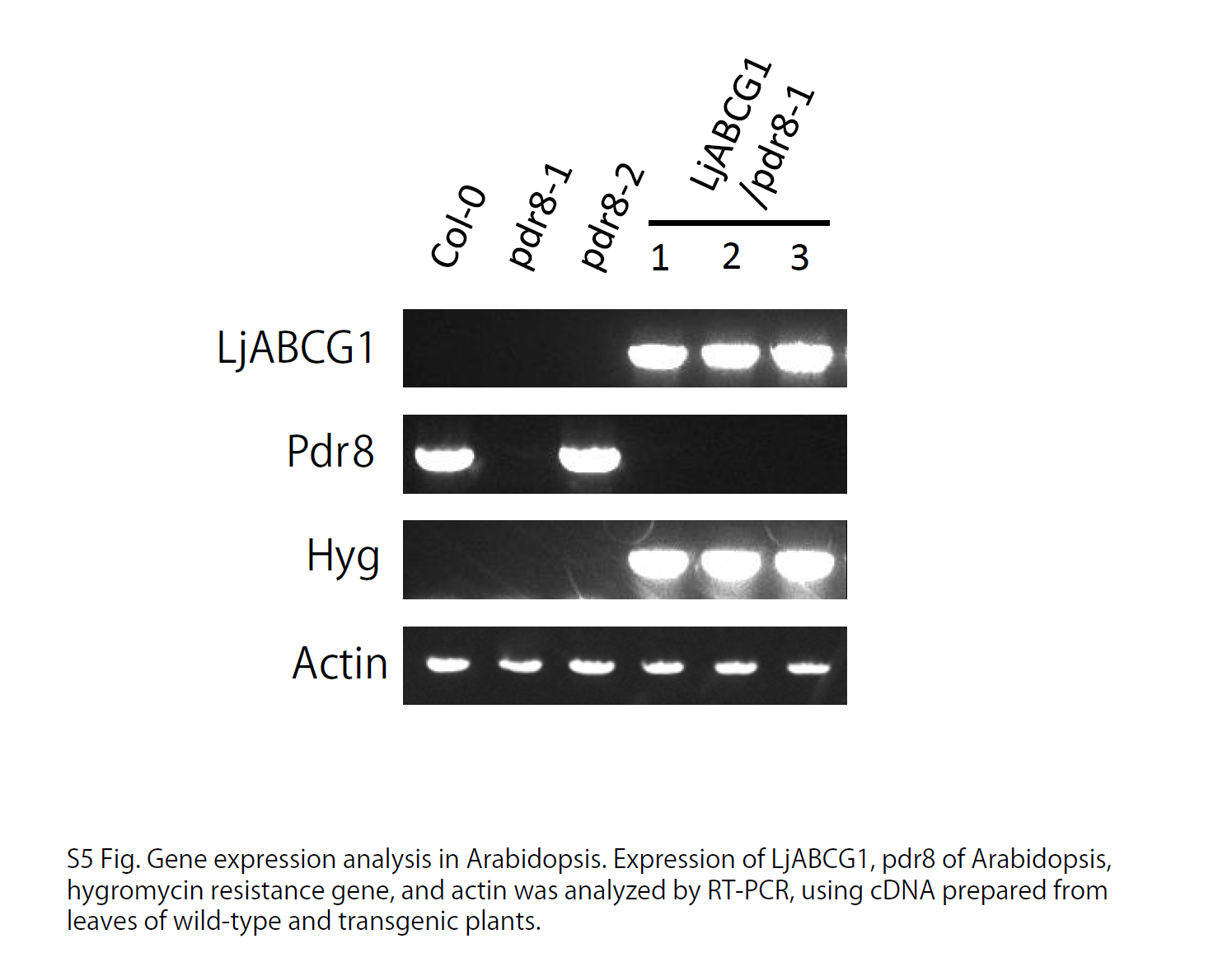

Supplement: S5 Fig — Expression of LjABCG1, pdr8 of Arabidopsis, hygromycin resistance gene, and actin was analyzed by RT-PCR, using cDNA prepared from leaves of wild-type and transgenic plants. (TIF) [file pone.0139127.s005.tif]

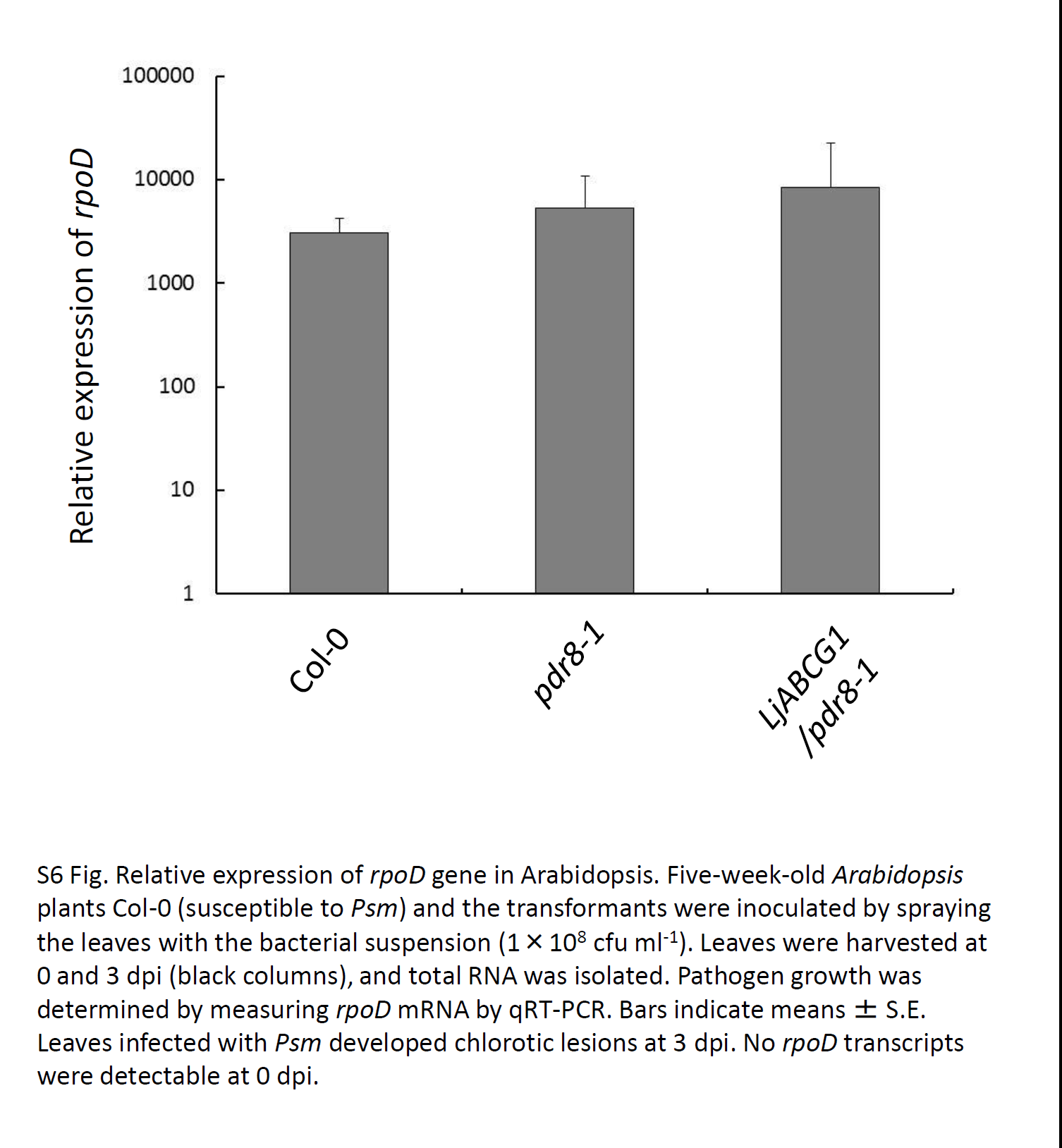

Supplement: S6 Fig — Five-week-old Arabidopsis plants Col-0 (susceptible to Psm) and the transformants were inoculated by spraying the leaves with the bacterial suspension (1×10^8^ cfu ml-1). Leaves were harvested at 0 and 3 dpi (black columns), and total RNA was isolated. Pathogen growth was determined by measuring rpoD mRNA by qRT-PCR. Bars indicate means ± S.E. Leaves infected with Psm developed chlorotic lesions at 3 dpi. No rpoD transcripts were detectable at 0 dpi. (TIF) [file pone.0139127.s006.tif]

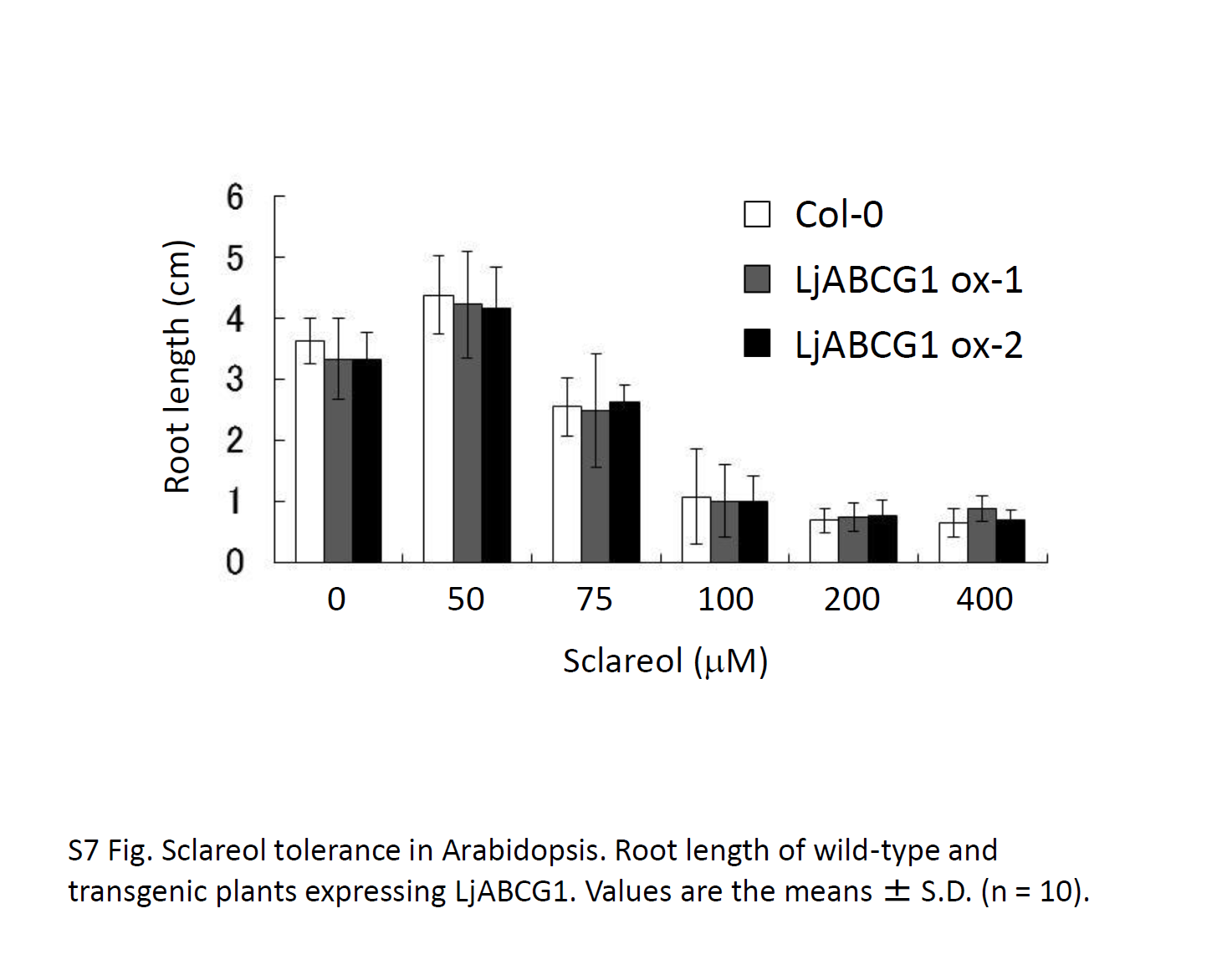

Supplement: S7 Fig — Root length of wild-type and transgenic plants expressing LjABCG1. Values are the means ± S.D. (n = 10). (TIF) [file pone.0139127.s007.tif]

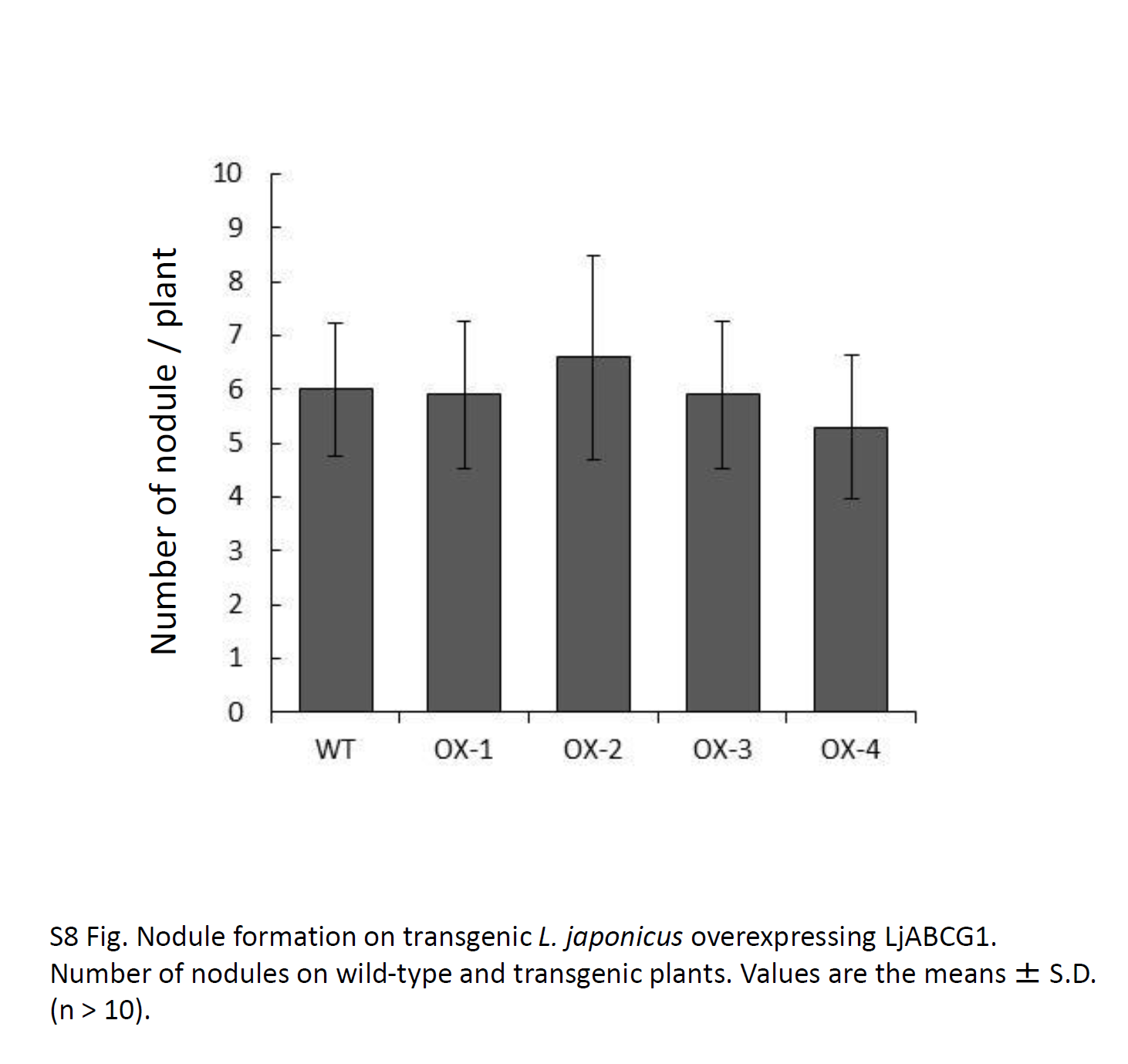

Supplement: S8 Fig — Number of nodules on wild-type and transgenic plants. Values are the means ± S.D. (n > 10). (TIF) [file pone.0139127.s008.tif]
