# Supplementary material for: Molecular Characterization of LjABCG1, an ATP-Binding Cassette Protein in Lotus japonicus
Source: PLoS One. 2015 Sep 29;10(9):e0139127. doi: 10.1371/journal.pone.0139127 (PMC4587964; doi:10.1371/journal.pone.0139127)
Supplement: S1 Table — (DOCX) [file pone.0139127.s009.docx]

| Supplementary Table 1. Plant hormone analysis in Arabidopsis leaves | | | | | | | | | | | |
| --- | --- | --- | --- | --- | --- | --- | --- | --- | --- | --- | --- |
|  | Col-0 | | |  | *pdr8-1* | | |  | LjABCG1/*pdr8-1* | | |
| tZ | 0.39 | ± | 0.05 |  | 0.35 | ± | 0.06 |  | 0.41 | ± | 0.07 |
| tZR | 0.10 | ± | 0.01 |  | 0.08 | ± | 0.03 |  | 0.09 | ± | 0.04 |
| tZRPs | 1.29 | ± | 0.33 |  | 1.06 | ± | 0.17 |  | 0.99 | ± | 0.36 |
| cZ | 0.18 | ± | 0.01 |  | 0.16 | ± | 0.03 |  | 0.18 | ± | 0.03 |
| cZR | 0.05 | ± | 0.00 |  | 0.07 | ± | 0.03 |  | 0.06 | ± | 0.02 |
| cZRPs | 0.72 | ± | 0.18 |  | 0.74 | ± | 0.17 |  | 0.64 | ± | 0.08 |
| DZ |  | nd |  |  | 0.03 | ± | - |  | 0.02 | ± | - |
| DZR |  | nd |  |  |  | nd | - |  |  | nd |  |
| DZRPs |  | nd |  |  | 0.02 | ± | - |  |  | nd |  |
| iP | 0.08 | ± | 0.01 |  | 0.10 | ± | 0.04 |  | 0.10 | ± | 0.01 |
| iPR | 0.02 | ± | 0.00 |  | 0.03 | ± | 0.00 |  | 0.03 | ± | 0.01 |
| iPRPs | 4.35 | ± | 0.51 |  | 5.09 | ± | 0.79 |  | 4.71 | ± | 1.39 |
| tZ7G | 35.38 | ± | 2.92 |  | 35.48 | ± | 5.45 |  | 36.98 | ± | 0.72 |
| tZ9G | 21.11 | ± | 0.16 |  | 21.55 | ± | 5.16 |  | 19.69 | ± | 0.95 |
| tZOG | 2.99 | ± | 0.34 |  | 2.57 | ± | 0.33 |  | 3.17 | ± | 0.87 |
| cZOG | 3.07 | ± | 0.69 |  | 3.08 | ± | 0.46 |  | 3.89 | ± | 1.20 |
| tZROG | 0.69 | ± | 0.09 |  | 0.54 | ± | 0.02 |  | 0.67 | ± | 0.14 |
| cZROG | 2.27 | ± | 0.48 |  | 2.03 | ± | 0.21 |  | 2.45 | ± | 0.66 |
| tZRPsOG | 0.02 | ± | 0.01 |  | 0.02 | ± | 0.01 |  | 0.02 | ± | 0.01 |
| cZRPsOG | 0.03 | ± | 0.00 |  | 0.02 | ± | 0.00 |  | 0.03 | ± | 0.01 |
| DZ9G |  |  |  |  | 0.05 | ± | - |  | 0.05 | ± | - |
| iP7G | 15.54 | ± | 0.84 |  | 15.81 | ± | 2.00 |  | 18.53 | ± | 2.82 |
| iP9G | 2.35 | ± | 0.76 |  | 3.11 | ± | 0.46 |  | 3.99 | ± | 1.02 |
|  |  |  |  |  |  |  |  |  |  |  |  |
| GA1 |  | nd |  |  |  | nd |  |  |  | nd |  |
| GA4 | 7.82 | ± | - |  | 14.29 | ± | - |  | 13.97 | ± | 2.47 |
| GA7 |  | nd |  |  |  | nd |  |  |  | nd |  |
| GA8 | 0.55 | ± | - |  | 0.50 | ± | - |  |  | nd |  |
| GA9 |  | nd |  |  |  | nd |  |  |  | nd |  |
| GA19 | 1.42 | ± | 0.23 |  | 1.50 | ± | 0.09 |  | 1.34 | ± | 0.27 |
| GA20 |  |  |  |  | 0.35 | ± | - |  | 0.66 | ± | - |
| GA24 | 3.04 | ± | 1.41 |  | 4.94 | ± | 1.37 |  | 5.58 | ± | 0.64 |
| GA44 |  | nd |  |  |  | nd |  |  |  | nd |  |
| GA53 | 1.38 | ± | - |  | 1.17 | ± | - |  |  | nd |  |
| IAA | 3314.92 | ± | 1659.80 |  | 4715.93 | ± | 1696.06 |  | 4792.93 | ± | 1119.67 |
| IAAla |  | nd |  |  |  | nd |  |  |  | nd |  |
| IAAsp | 10.62 | ± | 4.77 |  | 12.28 | ± | 4.31 |  | 7.60 | ± | 3.86 |
| IAIle+IALeu |  | nd |  |  |  | nd |  |  |  | nd |  |
| IAPhe |  | nd |  |  |  | nd |  |  |  | nd |  |
| IATrp |  | nd |  |  |  | nd |  |  | 14.20 | ± | - |
|  |  |  |  |  |  |  |  |  |  |  |  |
| SA | 5023.47 | ± | 282.57 |  | 19068.79 | ± | 15931.92 |  | 20876.87 | ± | 19184.57 |
| JA | 183.16 | ± | 119.73 |  | 383.36 | ± | 457.47 |  | 197.07 | ± | 93.16 |
| ABA | 13.47 | ± | 1.36 |  | 13.55 | ± | 1.79 |  | 16.31 | ± | 1.59 |
| nd, not detected; tZ, trans-zeatin; tZR, tZ riboside; tZRPs, tZR phosphates; cZ, cis-zeatin; cZR, cZ riboside; cZRPs, cZR phosphates; DZ, dihydrozeatin; DZR, DZ riboside; DZRPs, DZR phosphates; iP, N6-(Δ2-isopentenyl)adenine; iPR, iP riboside; iPRPs, iPR phosphates; tZ7G, tZ-7-N-glucoside; tZ9G, tZ-9-N-glucoside; tZOG, tZ-O-glucoside; tZROG, tZR-O-glucoside; cZOG, cZ-O-glucoside; cZROG, cZR-O-glucoside; cZRPOG, cZR phosphate-O-glucoside; tZRPOG, tZR phosphate-O-glucoside; DZ9G, DZ-9-N-glucoside; iP7G, iP-7-N-glucoside; iP9G, iP-9-N-glucosid; IAA, indole-3-acetic acid; IA-Ala, indole-3-acetyl-l-Ala; IA-Asp, indole-3-acetyl-l-Asp; IA-Leu, indole-3-acetyl-l-Leu; IA-Ile, indole-3-acetyl-l-Ile; IA-Phe, indole-3-acetyl-l-Phe; IA-Trp, indole-3-acetyl-l-Trp; SA, salicylic acid; JA, jasmonic acid; ABA, abscisic acid. - in S.D. indicates that one of the replicates showed nd. | | | | | | | | | | | |
|  |  |  |  |  |  |  |  |  |  |  |  |
|  |  |  |  |  |  |  |  |  |  |  |  |
|  |  |  |  |  |  |  |  |  |  |  |  |
|  |  |  |  |  |  |  |  |  |  |  |  |
|  |  |  |  |  |  |  |  |  |  |  |  |
|  |  |  |  |  |  |  |  |  |  |  |  |
|  |  |  |  |  |  |  |  |  |  |  |  |
|  |  |  |  |  |  |  |  |  |  |  |  |
